# Ferric Uptake Regulator Provides a New Strategy for Acidophile Adaptation to Acidic Ecosystems

**DOI:** 10.1128/AEM.00268-20

**Published:** 2020-05-19

**Authors:** Xian-ke Chen, Xiao-yan Li, Yi-fan Ha, Jian-qiang Lin, Xiang-mei Liu, Xin Pang, Jian-qun Lin, Lin-xu Chen

**Affiliations:** a State Key Laboratory of Microbial Technology, Shandong University, Qingdao, People’s Republic of China; University of Illinois at Urbana-Champaign

**Keywords:** ferric uptake regulator, *Acidithiobacillus*, acidophiles, acid resistance

## Abstract

Acidophiles play a dominant role in driving elemental cycling in natural acid mine drainage (AMD) habitats and exhibit important application value in bioleaching and bioremediation. Acidity is an inevitable environmental stress and a key factor that affects the survival of acidophiles in their acidified natural habitats; however, the regulatory strategies applied by acidophilic bacteria to withstand low pH are unclear. We identified the significance of the ferric uptake regulator (Fur) in acidophiles adapting to acidic environments and discovered that Fur is ubiquitous as well as highly conserved in acidophilic bacteria. Mutagenesis of the *fur* gene of Acidithiobacillus caldus, a prototypical acidophilic sulfur-oxidizing bacterium found in AMD, revealed that Fur is required for the acid resistance of this acidophilic bacterium. Phenotypic characterization, transcriptome sequencing (RNA-seq), mutagenesis, and biochemical assays indicated that the Acidithiobacillus caldus ferric uptake regulator (AcFur) is involved in extreme acid resistance by regulating the expression of several key genes of certain cellular activities, such as iron transport, biofilm formation, sulfur metabolism, chemotaxis, and flagellar biosynthesis. Finally, a Fur-dependent acid resistance regulatory strategy in *A. caldus* was proposed to illustrate the ecological behavior of acidophilic bacteria under low pH. This study provides new insights into the adaptation strategies of acidophiles to AMD ecosystems and will promote the design and development of engineered biological systems for the environmental adaptation of acidophiles.

**IMPORTANCE** This study advances our understanding of the acid tolerance mechanism of *A. caldus*, identifies the key *fur* gene responsible for acid resistance, and elucidates the correlation between *fur* and acid resistance, thus contributing to an understanding of the ecological behavior of acidophilic bacteria. These findings provide new insights into the acid resistance process in *Acidithiobacillus* species, thereby promoting the study of the environmental adaptation of acidophilic bacteria and the design of engineered biological systems.

## Abstract (precis)

This study advances our understanding of the acid tolerance mechanism of *A. caldus*, identifies the key *fur* gene responsible for acid resistance, and elucidates the correlation between *fur* and acid resistance, thus contributing to an understanding of the ecological behavior of acidophilic bacteria. These findings provide new insights into the acid resistance process in *Acidithiobacillus* species, thereby promoting the study of the environmental adaptation of acidophilic bacteria and the design of engineered biological systems.

## INTRODUCTION

Acidophiles are the main drivers for the formation of acid mine drainage (AMD) in natural habitats. Studies in the last several decades have shown that microorganisms in AMD ecosystems present considerable diversity and a high richness of acidophilic taxa (1, 2). Generally, *Proteobacteria*, *Acidithiobacillia*, *Nitrospira*, *Firmicutes*, *Acidobacteria*, *Actinobacteria*, *Aquificae*, *Euryarchaeota*, and *Crenarchaeota* are ubiquitous in AMD ecosystems (3, 4). Knowledge of the microorganisms promoting the material and energy flow of AMD ecosystems provides basic clues for AMD bioleaching and bioremediation, such as the oxidation of iron and sulfur by the acidophilic chemolithoautotrophic microbes, accelerating the biogeochemical cycles of elements and the release of metals (1, 4, 5). In addition, AMDs, similar to other metal-bearing wastes, are a potential secondary source for recovering metals. By using acidophiles in AMD, the biomining industry has developed new technology for extracting metals from ores through microbial oxidation (6). Moreover, acidophile-dependent bioleaching technology has been applied to the treatment of waste containing heavy metals, such as sewage sludge, spent household batteries, mine tailings, and printed circuit boards (7). In AMDs and bioleaching ecosystems, one of the main challenges to these microorganisms is extremely acidic stress (6–8). Thus, a comprehensive understanding of the acid resistance mechanisms of acidophiles would be helpful for the development of the AMD bioleaching industry and bioremediation.

Acidity is the major determinant of microbial community composition in AMD ecosystems. Although species diversity significantly decreases as the pH decreases, high richness of acidophilic taxa, such as *Gammaproteobacteria* and *Nitrospira*, was detected in AMD (9, 10). Members of the proteobacterial class *Acidithiobacillia* are prevalent in AMD and biomining environments because of the ability of these microbes to utilize sulfur and iron and adapt to extremely acidic environments (4). In these environments, *Acidithiobacillia* can dramatically accelerate the acid generation process; therefore, strains in this class play important roles in the material and energy flow of the AMD ecosystem (4). Moreover, several species of *Acidithiobacillia*, such as Acidithiobacillus caldus, Acidithiobacillus ferrooxidans, and Acidithiobacillus thiooxidans, have been found to be the most active and dominant bacteria in bioleaching applications (11). Therefore, the acid resistance of acidophiles is important for these microbes to survive and thrive in extremely acidic environments, thus determining their ability to occupy the ecological niche in these systems.

Microorganisms have evolved diverse acid resistance mechanisms and models to prevent cell damage due to acid stress (12–15), such as the efflux of protons (16), proton consumption (17), adjustment of cell membrane composition (18), DNA and protein repair systems (19), generation of reversed transmembrane electrical potential (Δψ) (20), and alkali production (21). However, most acid resistance mechanisms and models of acidophiles are limited to transcriptome, proteome, and bioinformatic analyses (22–25). Extremely acidophilic bacteria use a wide variety of strategies similar to those of neutralophiles for acid resistance that facilitate defense against extreme acid challenges, including more cation transporters and a large number of DNA and protein repair systems (12). Some global regulators are also reported to be involved in the regulation of cytoplasmic proton homeostasis, such as OmpR, RpoS, and Fur in most bacteria (14, 22).

The ferric uptake regulator (Fur), originally identified as a regulator of iron homeostasis in a variety of bacteria, has been reported to be involved in the regulation of the acid stress response in some neutralophiles, such as Salmonella enterica (26), Helicobacter pylori (27), and Campylobacter jejuni (28). Generally, Fur follows the classic regulation pattern in which *holo*-Fur binds to the Acidithiobacillus caldus ferric uptake regulator (AcFur) box in the promoter to restrict the binding of RNA polymerase and represses gene transcription (29). Moreover, Fur is involved in regulating a wide variety of crucial physiological metabolic pathways, including DNA synthesis (30), the tricarboxylic acid (TCA) cycle (31), biofilm formation (32), and expression of virulence genes, as well as the expression of some microbial genes responsible for the maintenance of iron homeostasis and defense against oxidative stresses (33, 34), in most prokaryotes. In general, the maintenance of cytoplasmic iron levels is regulated by Fur, except in a few Gram-positive and acid-resistant bacteria utilizing the diphtheria toxin repressor (DtxR) to regulate iron homeostasis, such as Corynebacterium diphtheriae (35). The functions of Fur in maintaining iron homeostasis and the response to acid resistance in A. ferrooxidans have been investigated (22, 36); however, the role of Fur in acid-adapted regulation in *Acidithiobacillus* spp. and the universality of the Fur-dependent regulation in acidophiles are still unclear.

Here, we analyzed the distribution of Fur in acidophiles and compared the sequence and structural features of Fur proteins in acidophilic bacteria. By using A. caldus (MTH-04) as a model, we investigated the AcFur and its role in adaptation to low pH. Fur-regulated phenotypes and genes of *A. caldus* were determined. Finally, the existence of a Fur-dependent acid adaptation strategy in *A. caldus*, as well as the significance of Fur for acid resistance regulation in acidophiles, was proposed.

## RESULTS

### Universality and conservation of Fur in AMD ecosystems.

To investigate the distribution of Fur in AMD bacteria, we first searched for AMD-related microorganisms and their genomes in the National Center for Biotechnology Information (NCBI) database according to previous reports (4, 5). We then determined an *e* value (<5.00E − 05) and searched for genome and metagenome sequences available in the NCBI database. Ultimately, the results based on the microorganism species, the assembly level of the microbial genome, the *e* value, and annotation were used for analysis (see Table S1 in the supplemental material). The results showed that about 89% of the AMD bacterial genomes contained *fur* genes. Interestingly, we found that the genus *Acidiphilium* (strictly aerobic; optimal pH range, 3 to 3.5; chemoorganotrophic bacteria) does not contain *fur* genes—for example, Acidiphilium cryptum JF-5 (genome accession number NC_009484.1), Acidiphilium multivorum AIU301 (genome accession number NC_015186.1), Acidiphilium angustum ATCC 35903 (genome accession number NZ_JNJH00000000.1), and Acidiphilium rubrum (genome accession number NZ_FTNE00000000.1). This discovery implied that harboring Fur is a rule rather than an exception in the bacteria of AMD ecosystems. Interestingly, Fur homologs are rarely found in most archaea of AMD communities, except for Thermoplasma volcanium GSS1 and Cuniculiplasma divulgatum PM4. However, most archaea possess the DtxR protein, which is similar to Fur in both protein structure and potential function (see Table S1) (37).

### The prominent role of AcFur in the extreme acid resistance of *A. caldus*.

**FIG 1:**
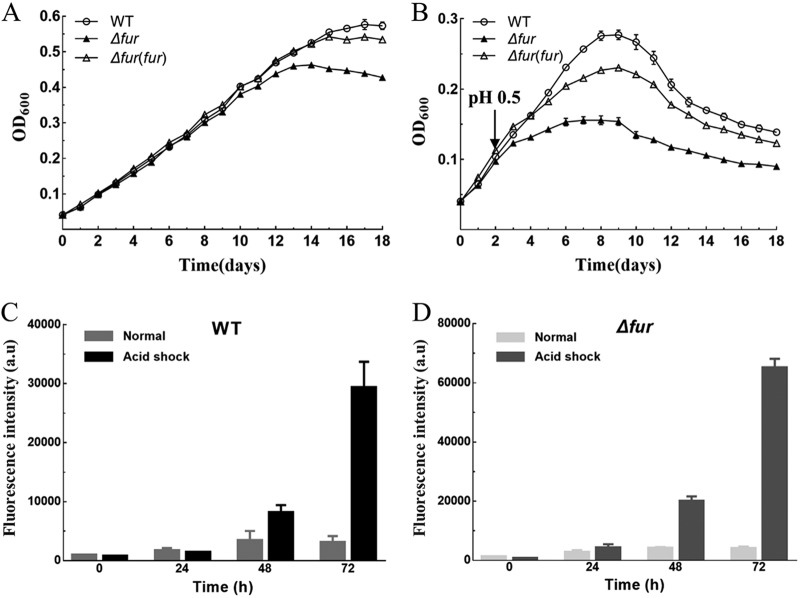
The *A. caldus fur* gene is involved in acid resistance. (A and B) Analyses of the growth curves of the *A. caldus* wild type (WT) and Δ*fur* strains grown in the Starkey-S^0^ medium. (A) Strains were grown under normal conditions. (B) After 2 days of cultivation in Starkey-S^0^ medium, sulfuric acid (1:1) was added to adjust the pH to 0.5. (C and D) The levels of *fur* expression in *A. caldus* (WT and Δ*fur*) before and after acid shock were detected by luciferase activity. Error bars show standard deviations.

To illustrate the role of Fur in AMD bacteria, *fur* deletion (*Δfur*) and *fur*-complemented [*Δfur*(*fur*)] strains of *A. caldus* were constructed using markerless knockout/knock-in technology. The *Δfur* strain exhibited an obvious growth disadvantage at the stationary phase compared with the wild-type (WT) and *Δfur*(*fur*) strains of *A. caldus* (Fig. 1A). The acid shock assay showed that the cell density of the *Δfur* strain was much lower than that of the WT and *Δfur*(*fur*) strains when the pH of the cultures was adjusted to 0.5 on the second day (Fig. 1B). Thus, the results identified the essential role of Fur in the response of *A. caldus* to low pH. Furthermore, a promoter-probe vector using the reporter luciferase gene (*luc*) was generated to monitor the expression level of *fur* under low pH conditions. When the WT strain was used as the host, acid shock caused a significant increase in the luciferase activity at 48 and 72 h after stimulation compared with the nonstimulated group (Fig. 1C). A similar phenomenon appeared in the host *Δfur* strain (Fig. 1D). These results suggested that the expression level of *fur* is closely related to the pH of the environment. Moreover, under the condition of acid shock, the luciferase activity of the *fur* mutant was twice that of the WT strain at 48 and 72 h after stimulation (Fig. 1C and D), indicating that Fur also has an effect on its own expression level in *A. caldus* when responding to low pH.

### Role of the key domains of Fur in acid resistance of *A. caldus*.

The amino acid sequences of the Fur proteins from acidophiles showed high similarity and conservation in the N-terminal DNA-binding domain, C-terminal dimerization domain, and metal-binding site motifs (29, 38). All Fur sequences of AMD bacteria exhibited a C-terminal extension, in contrast to some neutrophilic bacteria, but the sequence of this C-terminal extension is not conserved in these acidophilic bacteria (see Fig. S2). Thus, the evolutionary conservation of the sequence and structure of Fur proteins in acidophilic bacteria indicates that this protein might play similar roles in AMD bacteria adapting to acidic environments.

**FIG 2:**
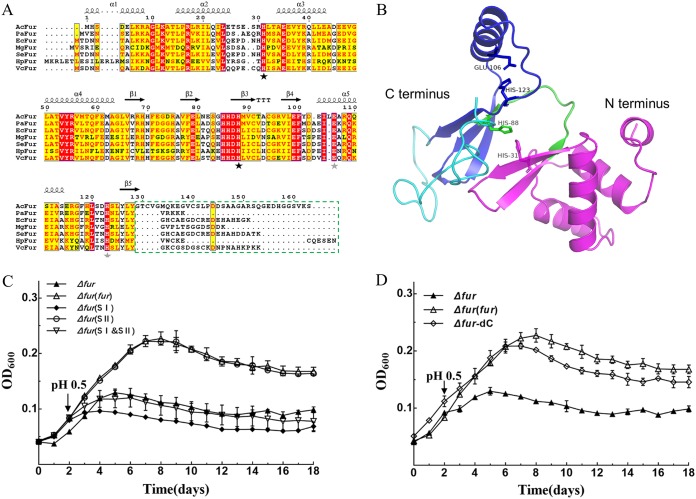
*In silico* analysis and functional confirmation of AcFur. (A) Multiple-sequence alignment of AcFur with Fur proteins from different bacteria by T-COFFEE and ESPript. The key residues of the metal-binding sites I and II are marked with black stars and gray stars under the sequences, respectively. The C-terminal extension of AcFur that was deleted to estimate its functionality is highlighted with a dashed box. (B) The AcFur model was generated by Phyre2 and visualized with PyMOL 2.0, as described in Materials and Methods. The model of the AcFur structure is shown in cartoon mode (N-terminal, magenta; C terminus, blue; linker, green; C-terminal extension, cyan). The putative key residues are shown as a stick model. (C and D) Phenotypic characterization of Δ*fur*, Δ*fur*(*fur*), Δ*fur*(SI), Δ*fur*(SII), Δ*fur*(SI&SII), and Δ*fur*-dC strains under acid shock. Error bars show standard deviations.

To compare and identify the key domains of Fur in *A. caldus*, an alignment of amino acid sequences of AcFur and modeling of its three-dimensional structure of homology were performed (Fig. 2A and B). Based on the results of multiple sequence alignment, evolutionary analysis, and structural comparison, the key residues (H31, H88, E106, and H123) of AcFur in the binding sites were found to be highly conserved in all orthologs. We then suggested that H31 and H88 constitute the regulatory site (SI domain) responsible for binding to the DNA sequence, and the other two key residues constitute the structural site (SII domain) responsible for the steady conformation (38, 39). The growth analysis showed that the mutation of SI (H31A and H88A) rendered the cells sensitive to low pH, while the substitution of the two key residues in SII (E106A and H123A) did not influence the growth of the *Δfur*(SII) strain (Fig. 2C). This result indicated that the SI domain is crucial for the maintenance of AcFur function in acid resistance. In addition, to test the role of the sequence of the AcFur C-terminal extension, a complemented Δ*fur*-dC strain lacking the C-terminal extension was constructed. The data showed that the deletion of the C-terminal extension of AcFur resulted in a lower cell density of the Δ*fur*-dC strain after 4 days of stimulation (Fig. 2D).

### Fur-regulated genes in *A. caldus* under acid shock.

**FIG 3:**
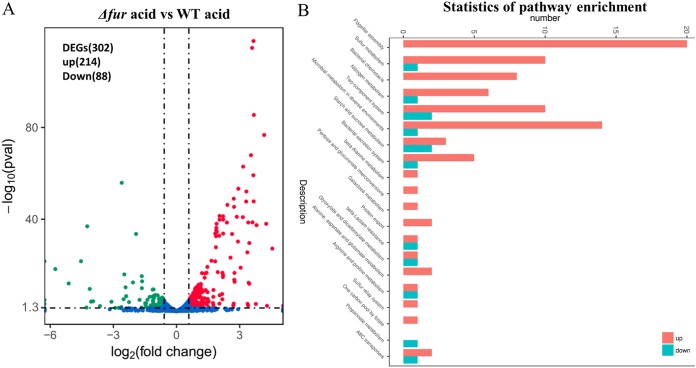
Overall transcriptomic changes in the Δ*fur* mutant during acid shock. (A) Volcano plot showing fold changes and levels of significance for differentially expressed genes. (B) KEGG pathway enrichment.

To further explore the Fur-mediated acid resistance mechanisms, transcriptome sequencing (RNA-seq) was performed to detect the differentially expressed genes (DEGs) in the *Δfur* strain after 48 h of acid shock. Overall, a total of 302 genes were differentially expressed in the *Δfur* mutant compared with the WT strain, including 214 upregulated and 88 downregulated genes (Fig. 3A). The KEGG pathway enrichment of the DEGs indicated that some pathways were influenced by the deletion of *fur* in *A. caldus*, and these were mainly related to bacterial chemotaxis, flagellar assembly, sulfur metabolism, nitrogen metabolism, and two-component systems (Fig. 3B). These results suggested that AcFur plays an important role in the response and adaptation of *A. caldus* to low pH and may affect a variety of physiological processes in *A. caldus*.

The deletion of the *fur* gene in *A. caldus* MTH-04 resulted in significant downregulation of genes involved in cell wall and membrane synthesis, biofilm formation, and the tetrathionate intermediate thiosulfate oxidation (S_4_I) pathway, such as the indirectly regulated Pel exopolysaccharide genes *pelA* (A5904_RS00180), *pelB* (A5904_RS00195), and *pelC* (A5904_RS00200) (40); the cell wall and membrane synthesis genes *bcsZ* (A5904_RS08000) and *bcsA* (A5904_RS08015); a gene (A5904_RS00585) encoding WbeA; the tetrathionate hydrolase gene *tetH* (A5904_RS03355); and the thiosulfate:quinol oxidoreductase gene *doxDA* (A5904_RS03360). The absence of the *fur* gene resulted in the upregulation of some genes participating in iron transport and direct regulation, including the *feoABC* operator (A5904_RS00865-00875), *mntH* (A5904_RS04110), and *feoP* (A5904_RS13315); the flagellar synthesis and chemotaxis genes *flhF* (A5904_RS04315), *flhA* (A5904_RS04320), *fliP* (A5904_RS04340), *fliG* (A5904_RS04380), *cheY* (A5904_RS04540), and *cheV* (A5904_RS04565), etc.; the sulfur-oxidizing enzyme (Sox) genes *ccsB* (A5904_RS10135), *soxA* (A5904_RS10140), *soxZ* (A5904_RS10175), *soxY* (A5904_RS10180), *soxX* (A5904_RS10925), and *soxB* (A5904_RS10950), etc.; and the nitrogen metabolism genes *ntrC* (A5904_RS02385), *nasA* (A5904_RS06340), and *nirD* (A5904_RS06365), etc., in *A. caldus* MTH-04 (see the supplemental DEG list [*fur* versus WT] in Data Set S1). In summary, AcFur affected the expression of a variety of crucial physiological genes, suggesting that for *A. caldus*, the Fur-dependent regulatory network is an essential adaptive mechanism for successfully coping with an extremely acidic environment.

### Regulation of iron transporter genes by Fur.

**FIG 4:**
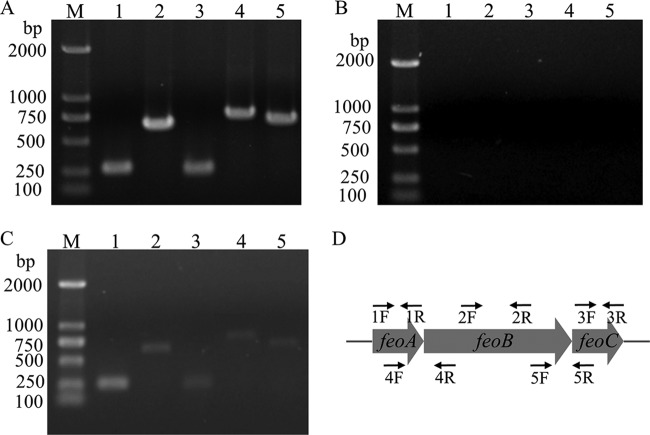
Cotranscriptional analyses of the *feoABC* cluster by reverse transcription-PCR. The templates for the PCR were genomic DNA (gDNA) (A), RNA removed from gDNA (B), and cDNA (C). Lanes M, 250-bp IDNA marker; lanes 1 through 5 are numbered with the sample number, corresponding to the primer number. (D) Locations of the primers used.

**FIG 5:**
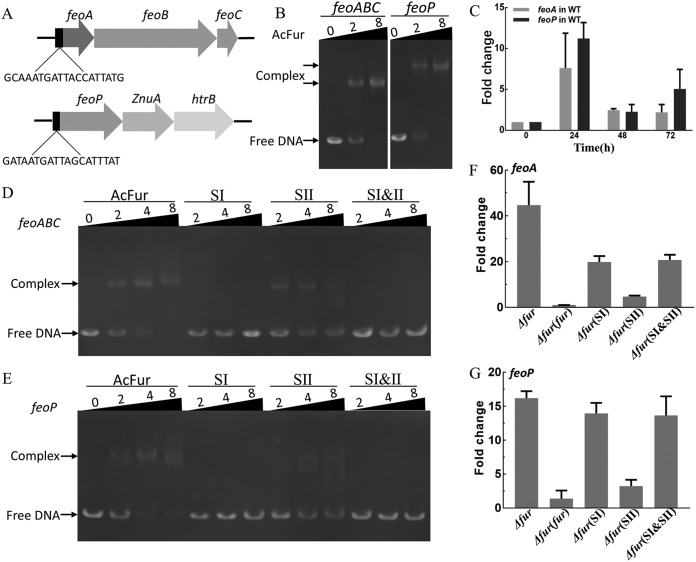
AcFur SI is important for iron transport. (A) The AcFur box of *A. caldus* MTH-04, predicted by using Virtual Footprint and gene locations. (B) Binding of AcFur to the *feoABC* operator and the *feoP* promoter at different concentrations of AcFur. (C) Expression of *feoA* and *feoP* genes in wild-type *A. caldus* strain monitored by qRT-PCR after acid shock. (D and E) Binding of the *feoABC* operator and the *feoP* promoter, respectively, to the AcFur, SI, SII, and SI plus SII at different concentrations of protein. (F and G) Expression levels of *feoA* and *feoP* in the *fur* knockout strain and *Δfur*(*fur*), *Δfur*(SI), *Δfur*(SII), and *Δfur*(SI&SII) strains, tested separately by qRT-PCR. Error bars show standard deviations.

Some known genes and operons are involved in iron transport in *A. caldus*, such as *feoABC* and *feoP*. Unlike the *feo* operon (*feoPABC*) in *A. ferrooxidans*, the *feo* operon in *A. caldus* is composed of *feoABC* (Fig. 4), while *feoP* is located in another cluster (*feoP-znuA-htrB*) (Fig. 5A). The Fur binding sequences (AcFur box) were predicted upstream of both the *feo* operon and *feoP* genes (Fig. 5A). Gel shift assays showed that Fur could bind to the promoter regions of *feoA* and *feoP* (Fig. 5B). Reverse transcription-quantitative PCR (RT-qPCR) indicated that the transcriptional levels of these two genes in the *A. caldus* WT strain were significantly upregulated after acid shock (Fig. 5C). The mutation of the SI domain resulted in the loss of the ability of Fur to bind to the promoters of *feoA* and *feoP*, and the affinity became weaker when the SII domain was mutated (Fig. 5D and E). The absence of *fur* markedly increased the transcription of *feoA* and *feoP*, and the Fur-dependent regulatory effect was restored when *fur* was complemented with *Δfur* (Fig. 5F and G). The mutation of SI and SII also affected the Fur-dependent regulation of *feoA* and *feoP*. SI mutation resulted in the obvious upregulation of *feoA* and *feoP*, which is similar to the regulatory effect caused by the deletion of *fur*. The influence of the SII mutation on the regulatory effect was much weaker than that of the SI mutation (Fig. 5F and G). Therefore, Fur could directly bind to promoters of *feoA* and *feoP*, generating an inhibitory effect on the transcription of iron transport operons. These results from mutagenesis, phenotypic characterization, and function studies suggested that iron homeostasis and pH homeostasis of AcFur are not separable.

### Regulatory effect of Fur on biofilm formation and EPS generation.

**FIG 6:**
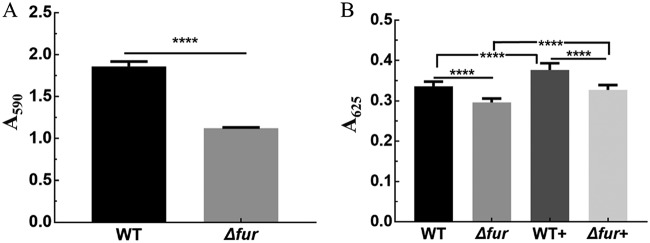
Disruption of *fur* reduces *A. caldus* MTH-04 biofilm formation and EPS synthesis. (A) Biofilm formation in sulfur coupons. (B) Carbohydrate levels in EPS, WT+, and Δ*fur*+ strains (+ indicates under acid shock). Error bars show standard deviations. ****, *P* < 0.0001.

The differential expression of genes involved in biofilm formation suggested the significance of biofilm in the adaptation of *A. caldus* to low pH; thus, we tested the influence of *fur* deletion on biofilm formation by using crystal violet staining. The data showed that the deletion of *fur* resulted in a decrease in biofilm formation (Fig. 6A). Furthermore, extracellular polymeric substances (EPS) were extracted by using heat treatment, and carbohydrate content in EPS was measured by the anthrone method to assess the effect of the *fur* gene knockout on EPS. Under acid shock conditions, both the WT and *fur* deletion strains showed significant increases in EPS production, suggesting the critical role of EPS synthesis in *A. caldus* adaptation to low pH. The amount of EPS in the Δ*fur* strain was lower than that in the WT both under normal and acidic shock conditions (Fig. 6B), suggesting that the absence of *fur* could affect EPS synthesis. Therefore, the significant decrease in EPS and biofilm caused by the deletion of *fur* suggested that AcFur is required for the regulation of biofilm formation and EPS generation under acid shock.

## DISCUSSION

In this work, the indispensable role of Fur in *A. caldus* adapting to low pH was uncovered, thus providing new insights into the adaptive mechanism of acidophiles to extremely acidic environments. Fur and DtxR homologs are ubiquitous in almost all acidophiles of AMD ecosystems (see Table S1), suggesting that both metal response regulators Fur and DtxR are essential for AMD acidophilic bacteria. Fur and DtxR do not exhibit any obvious sequence similarity. However, Fur is similar to DtxR in its structure, such as its DNA-binding domain (37). In addition, both of these proteins regulate iron homeostasis and some basic physiological and metabolic processes (35, 41). In some bacteria, such as Campylobacter jejuni (42) and Escherichia coli (43), Fur senses environmental stimuli and stresses and has a key function. The fact that many endosymbionts and those bacteria that live in cultures within nutrient-rich environments do not possess *fur* genes (44) suggests that the function of Fur is biased toward environmental response or adaptability. Our experimental evidence demonstrated that disruption of the *fur* gene reduces the acid tolerance of *A. caldus* and suggested the significance of Fur in acidophilic bacteria.

The sequences and structures of Fur proteins in AMD bacteria are highly conserved and homologous, but there are some differences from those in other bacteria. Unlike the Fur proteins of some neutrophilic bacteria, the nonconserved C-terminal extensions are universally present in the Fur proteins of AMD bacteria (Fig. 2D; also, see Fig. S2). The deletion of the C-terminal extension resulted in a decrease in the solubility of the recombinant protein (see Fig. S3), which altered the regulatory effect of Fur to some extent (Fig. 2D). In general, Fur proteins contain at least two metal-binding-site motifs, including functional and structural sites (29, 39, 45). We found that the SI domains (H31, H88) of AcFur in the metal-binding sites were highly conserved in all orthologs and confirmed that the SI domain is important for iron transport and acid resistance in *A. caldus* (Fig. 2A and C and Fig. 5D to G; also, see Fig. S2), suggesting that SI might be a functional site. In addition, the SII domain (E106, H123) is not strictly conserved in many bacteria, suggesting that SII probably plays an auxiliary role in *A. caldus* (Fig. 2A and C and Fig. 5D to G).

**FIG 7:**
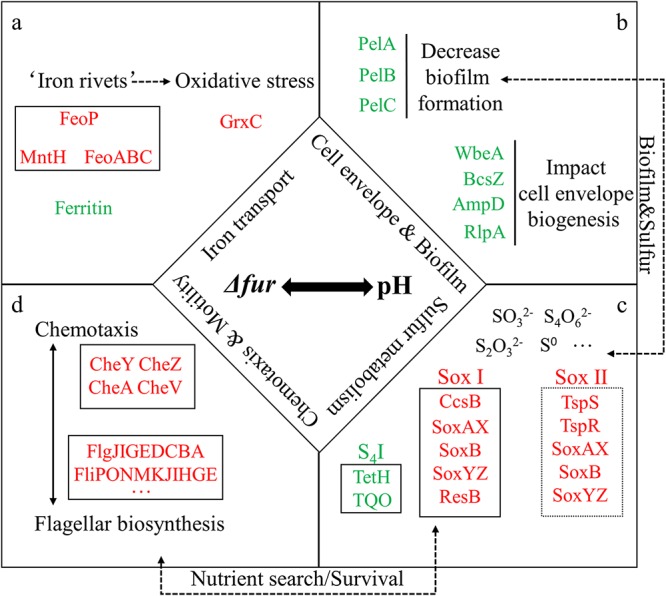
The AcFur regulatory network is involved in many required cellular functions in addition to iron acquisition. AcFur directly regulates genes associated with iron transport (a), cell envelope and biofilm (b), sulfur metabolism (c), and chemotaxis and motility (d). Red indicates upregulation, and green indicates downregulation. TQO, thiosulfate:quinol oxidoreductase.

Fur, as a global regulator, is directly or indirectly involved in a variety of crucial physiological and metabolic pathways in many bacteria (30, 32, 46). The RNA-seq data showed that 11.3% of genes were influenced by disruption of the *fur* gene in *A. caldus*, under the acid shock condition, including those involved in iron transport, chemotaxis and motility, biofilm formation, and energy system (Fig. 3). In addition, we showed that AcFur directly regulates iron transport and found that AcFur is important for *A. caldus* growth in an extremely acidic environment. Thus, it could be concluded that the Fur-dependent regulatory mode is probably a favored strategy used by acidophilic bacteria in response to acid stress (Fig. 7).

Most prokaryotic organisms can protect themselves from the environmental stress of low pH by forming biofilms and a complex cell envelope that consists of a plasma membrane, a peptidoglycan cell wall, and an outer membrane (15, 47). Some genes related to cell envelope synthesis were significantly downregulated, suggesting that cell envelope synthesis and components were affected, and this change might increase proton permeability in the Δ*fur* strain. Furthermore, the regulation of Fur on biofilm formation and EPS synthesis was confirmed by transcriptional and biochemical analyses (Fig. 6; also, see the supplemental DEG list [*fur* versus WT] in Data Set S1). Disruption of the *fur* gene reduced biofilm formation and EPS synthesis (Fig. 6), which explained the acid-sensitive phenotype of the Δ*fur* mutant to a certain degree (Fig. 1A and B). Flagellum-driven bacterial chemotaxis and motility are important for microorganisms responding to unfavorable environments (48, 49). Because of the important role of the EPS layer in bacterial elemental sulfur oxidation (50), the decrease in EPS synthesis observed in the Δ*fur* mutant probably reduced the available sulfur substrates and resulted in the adjustment of the two important periplasmic thiosulfate-metabolic pathways (the Sox system and the S_4_I pathway). Because of the close correlation between the Sox system and flagellum in *A. caldus* (51), the upregulation of the genes involved in these two systems suggested that the mutant strain had to adjust its metabolic flow to increase flagellum biosynthesis to escape unfavorable environments and find suitable habitats. Thus, the deletion of *fur* caused the readjustment of sulfur metabolism, flagellum biosynthesis, and chemotaxis, indicating the important regulatory role of Fur in *A. caldus* in response to low pH.

The acquisition of iron may be key to the survival of bacteria at low pH; however, high levels of intracellular free iron probably increase oxidative damage to cells (34, 52). It has been reported that iron transporters have been found in most acidophilic microorganisms and that these enzymes could be stabilized by “iron rivets” in acidophilic archaea (12). The transcription of *feoA* and *feoP* surged to a high level during acid stimulation (Fig. 5C), indicating that metal ion transport systems play a certain role in the acid stimulation process. Moreover, the direct regulation of iron transport genes by AcFur was also confirmed in *A. caldus*. These results suggested that Fur-regulated iron transport could affect the iron homeostasis of *A. caldus* under acid stimulation, which is a possible reason for the weak acid-adapted ability of the Δ*fur* and Δ*fur* (SI) strains (Fig. 1B and Fig. 2C). In addition, some common acid resistance genes related to l-glutamate (Glu), including those for the γ-aminobutyric acid (GABA) antiporter GadC and the Glu decarboxylase system, and DNA and protein repair system genes (13) were upregulated in the Δ*fur* mutant after the acid shock (see the supplemental DEG list [*fur* versus WT] in Data Set S1). This result implied that the enhanced activity of these systems could facilitate the survival of the Δ*fur* strain in an extremely acidic environment.

Acidity is not only a challenge for the application of acidophiles but also a basic clue for understanding the ecological behavior of AMD bacteria. Acidification occurs during bioleaching and bioremediation, which can seriously affect bacterial growth and application efficiency. The unique acid-resistant module of the acidophilic bacteria may be a potential target for application to engineering strains, with far-reaching potential applications (15, 53). The findings of our study provide new insights that would augment the current knowledge base pertaining to the acid resistance process in *Acidithiobacillus* species, and they could also help promote the study of the environmental adaptation of acidophilic bacteria and facilitate the design of engineered biological systems.

## MATERIALS AND METHODS

### Strains and culture conditions.

All bacterial strains, plasmids, and primers are listed in Table 1. *A. caldus* MTH-04 and its derivatives were grown in liquid Starkey-S^0^ inorganic medium (pH 2.5) or on solid Starkey-Na_2_S_2_O_3_ medium (pH 4.8). E. coli strains were grown in Luria-Bertani (LB) broth or on LB agar plates. Ampicillin (Amp), streptomycin (Sm), and kanamycin (Km) were used at 100 μg/ml in the LB medium, with the concentrations doubled in the Starkey medium. Chloramphenicol (Cm) was used at 60 μg/ml in the Starkey medium. The culture conditions were 40°C and 150 rpm for *A. caldus* MTH-04 and 37°C and 200 rpm for E. coli.

**TABLE 1:** Strains, plasmids, and primers

Strain, plasmid, or primer	Genotype description or sequence^*a*^	Reference or source
Strains		
*A. caldus* MTH-04	Isolated from Tengchong, Yunnan Province, China	Lab stock
*A. caldus* MTH-04 Δ*fur*	*fur* gene deletion	This study
*A. caldus* MTH-04 Δ*fur*(*fur*)	*fur* gene complementation for Δ*fur*	This study
*A. caldus* MTH-04 Δ*fur*(SI)	*fur* gene complementation for Δ*fur*; H31A H88A of *fur*	This study
*A. caldus* MTH-04 Δ*fur*(SII)	*fur* gene complementation for Δ*fur*; E106A H123A of *fur*	This study
*A. caldus* MTH-04 *Δfur*(SI&II)	*fur* gene complementation for Δ*fur*; H31A H88A E106A H123A of *fur*	This study
*A. caldus* MTH-04 Δ*fur*-dC	*fur* gene complementation for Δ*fur*; deletion of C-terminal extension of *fur*	This study
E. coli DH5α	F^−^ ϕ80d*lacZ*ΔM15 Δ(*lacZYA-argF*)*U169 end A1 recA1 hsdR17*(r_K_^−^ m_K_^+^) *supE44*λ-*thi-1 gyr96 relA1 phoA*	TransGen Biotech
E. coli SM10	*thr leu hsd recA* Km^r^ RP4–2-Tc::Mu	61
E. coli BL21(DE3)	F^−^ *ompT hdsSB*(r_B_^−^ m_B_^−^) *gal dgmmet* (DE3)	TransGen Biotech
Plasmids		
pSDUDI	Amp^r^ Km^r^; oriTRP4	Lab stock
pMSD1-I-Sce I	Sm^r^; mob^+^; *I-SceI* gene	Lab stock
pSDUDI∷*fur* (UHA+DHA)	Suicide plasmid for *fur* deletion	This study
pSDUDI∷*fur* (UHA+*fur*+DHA)	Suicide plasmid for *fur* complementation	This study
pSDUDI∷*fur* (UHA+*fur*(SI)+DHA)	Suicide plasmid for *fur* complementation; H31A H88A of *fur*	This study
pSDUDI∷*fur* (UHA+*fur*(SII)+DHA)	Suicide plasmid for *fur* complementation; E106A H123A of *fur*	This study
pSDUDI∷*fur* (UHA+*fur*(SI&II)+DHA)	Suicide plasmid for *fur* complementation; H31A H88A E106A H123A of *fur*	This study
pSDUDI∷*fur* (UHA+*fur*-dC+DHA)	Suicide plasmid for *fur* complementation; deletion of C-terminal extension of *fur*	This study
pJRD215-Luc-Cm	Sm^r^ Km^r^; IncQ Mob^+^; *luc cat*	Lab stock
pJRD215-P_fur_-Luc-Cm	Sm^r^ Km^r^; IncQ Mob^+^; *luc cat*; P*_fur_*	This study
pET-22b	Amp^r^	Novagen
pET-22b-Fur	Amp^r^; *fur*	This study
pET-22b-Fur(SI)	Amp^r^; H31A H88A of *fur*	This study
pET-22b-Fur(SII)	Amp^r^; E106A H123A of *fur*	This study
pET-22b-Fur(SI&II)	Amp^r^; H31A H88A E106A H123A of *fur*	This study
pET-22b-dC	Amp^r^; deletion of C-terminal extension of *fur*	This study
Primers		
*fur* UF-XbaI	TTCTAGGCTCTAGAGACAGGGAGCAGGAACG	This study
*fur* UR-SpeI	TTCTAGACTAGTCGGGTCGCGCACACCC	This study
*fur* DF-SpeI	TTCTAGACTAGTGAAACTCATGGCCAAAGTCTGA	This study
*fur* DR-HindIII	TTTCCCAAGCTTGTGGGTTCTGCCAATCTC	This study
F1	TCACGATTTGACCGAGCC	This study
R1	CCTCAAGGCCACGCTC	This study
F2	TTCAGCCTGGGTCTCG	This study
R2	CATTGGTCGGGGTGCC	This study
F3	ATCTGCATCCCCACTTCT	This study
R3	CACTTTTGCGCTTTGGTA	This study
D-F-*fur*	GCCAGCCCTTTTCAATTCATCGCTGTGCATGAAACTCATGGCCAAAGTCTGA	This study
U-R	CGGGTCGCGCACACCC	This study
*fur*-F-U	CCTGAGGCGCTCAGGGTGTGCGCGACCCGTCACGATTTGACCGAGC	This study
*fur*-R	ATGCACAGCGATGAATTG	This study
fH31A F	ACCAGCGAGAGCCGCGCTCTCACCGCCGAGGAG	This study
fH31A R	CTCCTCGGCGGTGAGAGCGCGGCTCTCGCTGGT	This study
fH88A F	AGCGGCCACCACGATGCCATGGTCTGCACCGCC	This study
fH88A R	GGCGGTGCAGACCATGGCATCGTGGTGGCCGCT	This study
fE106A F	TACGACGAGATCCTGGCCGCACGCCAGCAAAGC	This study
fE106A R	GCTTTGCTGGCGTGCGGCCAGGATCTCGTCGTA	This study
fH123A F	TTTCATCTCTCGGACGCTAGCCTCTATCTCTAC	This study
fH123A R	GTAGAGATAGAGGCTAGCGTCCGAGAGATGAAA	This study
P_fur_-F-MluI	CGACGCGT ATCGTCCTTGAACTGCA	This study
P_fur_-R-NdeI	GGAATTCCATATGGAAACTCATGGCCAAAG	This study
Fur F NdeI	GGAATTCCATATGATGCACAGCGATGAATTG	This study
Fur R XhoI	CCCTCGAGCGATTTGACCGAGC	This study
dFur R XhoI	CCCTCGAGTCAGGTTCCGTAGAGATAGA	This study
FeoABC-F	GCGTTTTACACCCAGGCAC	This study
FeoABC-R	TTCCACCTGCTCACCTGC	This study
FeoP-F	GCGTAGGACGTCCTTGATA	This study
FeoP-R	GCTGGAAGCAAGCATGGTG	This study
feoAF	CGCCGTCCTTCAAATCG	This study
feoAR	GTCATCCTGCCTGTTCCC	This study
feoPF	GGGATCCAAGCTCGGTATAT	This study
feoPR	CCACTTTCACGACATAGCCA	This study

a Underlining indicates restriction endonuclease sites.

### Construction of *fur* knockout and complementation strains.

The *fur* gene disruption (Δ*fur*) mutant was constructed using markerless knockout technology as described previously (54). To study the properties of the *fur* gene *in vivo*, *fur* complementation strains were constructed. To construct the complementation strains, markerless knock-in technology was applied to the Δ*fur* strains. First, the suicide plasmid pSDUDI::*fur* (UHA+*fur*+DHA) was constructed. The upstream and downstream homologous arms (UHA and DHA) and the *fur* gene were linked using fusion PCR. Next, single-crossover mutants were selected on solid Starkey-Na_2_S_2_O_3_ medium containing Km and identified by PCR. Finally, the pMSD1-I-SceI plasmid was transferred into the single crossover of *A. caldus* MTH-04 to induce the second homologous recombination, thereby generating the *fur* complementation strains. All plasmids, the Δ*fur* strain, and its derivatives of *A. caldus* MTH-04 were confirmed by PCR and DNA sequencing (Genewiz, Tianjin, China). The construction of the *fur* mutant complementation strains was performed as described above.

### Acid shock assay and transcriptional analysis.

The wild type (WT) and the Δ*fur* mutant of *A. caldus* MTH-04 were cultivated as described previously (54). Briefly, 1 ml of the treated cell culture (optical density at 600 nm [OD_600_] = 1.0) was inoculated into 150 ml of Starkey-S^0^ liquid inorganic medium (pH 2.5). After 3 days of cultivation, the pH of the culture was adjusted to 0.50 using H_2_SO_4_. Additionally, a culture without the addition of H_2_SO_4_ was used as the control. Total RNA was extracted 48 h after stimulation, and transcriptional analyses via RNA-seq and RT-qPCR were performed as described previously (51). The original analysis of DEGs is listed in the supplemental DEG list (*fur* versus WT) in Data Set S1.

### Expression analysis of AcFur using a luciferase reporter gene.

To investigate the expression levels of the *fur* gene in *A. caldus* responding to acid stress, the promoter region of *fur* (351 bp in front of the *fur* gene) from the *A. caldus* MTH-04 chromosome was amplified with the primer pair P_fur_-F-MluI–P_fur_-R-NdeI and inserted into the MluI- and NdeI-treated pJRD215-Luc-Cm plasmid containing the firefly luciferase (*luc*) gene and the chloramphenicol acetyltransferase (*cat*) gene. The generated pJRD215-P_fur_-Luc-Cm plasmid was conjugated into WT *A. caldus* MTH-04 and its Δ*fur* mutant. Luciferase activities were detected at 0, 24, 48, and 72 h after acid shock.

Luciferase activity was measured using a TransDetect single-luciferase (firefly) reporter assay kit. Brieﬂy, a culture of each strain was collected and diluted with phosphate-buffered saline (PBS; 10 mM PO_4_^3−^, 0.8% NaCl, pH 7.4) to an OD_600_ of 1. First, 2 ml of cells was washed three times with PBS buffer and harvested by centrifugation at 10,000 × *g* for 2 min at 4°C. Next, the cells were lysed by adding 200 μl of cell lysis buffer (1×) and incubating for 20 min at 20°C. Finally, after centrifugation at 13,000 × *g* for 15 min at 4°C, 100 μl of the supernatant was collected, and 90 μl of luciferase reaction reagent was added. Luciferase activity in the samples was measured using an EnSpire luminometer.

### Crystal violet biofilm assay and extracellular polymeric substance extraction.

*A. caldus* MTH-04 strains were incubated in 150 ml of Starkey-S^0^ medium for 8 days and then collected and diluted with Starkey liquid inorganic medium to an OD_600_ of 1.0. Subsequently, 1 ml of each type of bacterial cells was inoculated into 30 ml of sulfur coupon-containing Starkey medium. After static cultivation for 8 days, the sulfur coupons were washed gently three times with double-distilled water (ddH_2_O), dried for 40 min at 50°C, and stained with 1 ml of 1% (wt/vol) crystal violet (CV) solution for 15 min at room temperature. After staining, the sulfur coupons were washed with ddH_2_O and dried for 40 min at 50°C; 1 ml of 30% acetic acid was added to dissolve the CV, and the absorbance of the suspensions at 590 nm was quantitatively measured using a spectrophotometer (55).

Extracellular polymeric substances (EPS) was extracted using heat treatment (56). The EPS extraction method was adapted from that described by Xiao et al. (57). Briefly, the cells were harvested by centrifugation at 5,000 × *g* for 10 min at 4°C and washed twice with PBS buffer (10 mM PO_4_^3−^, 0.8% NaCl, pH 7.4). The washed cell pellets were resuspended in PBS buffer and heated in a water bath at 60°C for 30 min. After centrifugation at 12,000 × *g* for 30 min at 4°C, the supernatant was filtered using a 0.22-μm filter. Carbohydrate content was measured using the anthrone method (58).

### EMSA.

AcFur direct binding of the AcFur box was demonstrated by electrophoretic mobility shift assays (EMSA). Using the AcFur box of Pseudomonas aeruginosa as a pattern, the genome of *A. caldus* MTH-04 was searched with Virtual Footprint (59), and the potential AcFur box sequences were then selected based on the score. A 200-bp *feo* operator and a 190-bp *feoP* promoter containing the AcFur box were amplified from the *A. caldus* MTH-04 genome. PCR products were purified using a gel extraction kit (Omega), and 0.4 nM DNA was incubated with different concentrations of protein (0, 2, 4, and 8 nM) in Tris-HCl (10 mM, pH 8.0), NaCl (100 mM), and MnCl_2_ (1 mM) at room temperature for 30 min. The samples were separated in native polyacrylamide gels (5%) containing 0.5× Tris-borate (TBE) buffer and stained with ethidium bromide (EB).

### Generation and analysis of the three-dimensional AcFur structure.

The model of AcFur the three-dimensional structure was performed using the Phyre2 web portal (60) and using P. aeruginosa Fur (PDB code 1MZB), Vibrio cholerae Fur (PDB code 2W57), Magnetospirillum gryphiswaldense MSR-1 Fur (PDB code 4RAZ), E. coli Fur (PDB code 2FU4), Francisella tularensis Fur (PDB code 5NBC), Campylobacter jejuni Fur (PDB code 4ETS), etc., as templates. The protein structure model was refined using ModRefiner and evaluated using PSVS (Procheck [https://servicesn.mbi.ucla.edu/PROCHECK/], MolProbity Clashscore [http://molprobity.manchester.ac.uk/], Verify 3D [https://servicesn.mbi.ucla.edu/Verify3d/]) and SAVES v5.0 (Verify 3D, ERRAT, and PROVE [http://servicesn.mbi.ucla.edu/SAVES/]) online servers. The best protein model of AcFur was selected and visualized using PyMOL software (https://pymol.org/2/). Additionally, potential regulator sites in the AcFur sequences were assessed using T-COFFEE (http://tcoffee.crg.cat/) multiple sequence alignment.

### Statistical analysis.

All measurements were performed in triplicate, and all assays were repeated at least three times. Statistical analysis was performed using GraphPad Prism7.0 software. Significance was assessed by using an independent-samples *t* test.

### Data availability.

The *A. caldus* MTH-04 genome sequence and the raw RNA-seq data have been submitted to NCBI with the accession numbers PRJNA318365 and PRJNA577835, respectively. The strain *A. caldus* MTH-04 was isolated from Tengchong area, Yunnan Province, China, and has been deposited in the China General Microbiological Culture Collection Center (CGMCC) with the accession number CGMCC 1.15711. The mutants of *A. caldus* MTH-04 constructed in this study were stocked in our lab and will be made available upon request.

## Supplementary Material

Supplemental file 1

Supplemental file 2
