# Supplementary material for: Ferric Uptake Regulator Provides a New Strategy for Acidophile Adaptation to Acidic Ecosystems
Source: Appl Environ Microbiol. 2020 May 19;86(11):e00268-20. doi: 10.1128/AEM.00268-20 (PMC7237784; doi:10.1128/AEM.00268-20)
Supplement: Supplemental file 1 [file AEM.00268-20-s0001.pdf]

Supplemental Materials

Ferric uptake regulator provides a new strategy for acidophile adaptation to acidic ecosystems

Xian-ke Chen, Xiao-yan Li, Yi-fan Ha, Jian-qiang Lin, Xiang-mei Liu, Xin Pang, Jian-qun Lin*, and Lin-xu Chen*

State Key Laboratory of Microbial Technology, Shandong University, No. 72 Binhai Road, Qingdao 266237, P.R. China.

*** Correspondence:**

Lin-Xu Chen, Jian-Qun Lin

linxuchen@sdu.edu.cn, jianqunlin@sdu.edu.cn

Supplemental Materials and Methods

Plasmid construction, protein expression and purification

The *fur* gene was PCR-amplified from *A. caldus* MTH-04 chromosome and inserted into the *Nde* I and *Xho* I sites of pET22b and the resulting plasmid was transformed into *E. coli* DH5α cells. To study AcFur protein properties, three AcFur mutants (Site I (SI) H31A/H88A, Site II (SII) E106A/H123A, SiteI and SiteII (SI&SII) H31A/H88A/E106A/H123A) were generated by site-directed mutagenesis. All plasmids were confirmed by DNA sequencing (GENEWIZ, Tianjin, China).

E. coli BL21 Star harboring the *fur* and *fur* mutants plasmids were grown in LB medium of 0.01% $FeCl_2$ containing 100 μg/mL Amp until the OD_{600} = 0.8 and were induced by adding isopropyl β-D-1-thiogalactopyranoside (IPTG) (0.2 mM, final concentration) and incubating overnight at 18 ℃. The cells were harvested by centrifugation at 8000 ×g for 15 min at 4 ℃ and lysed by ultrasonication in lysis buffer (25 mM Tris-HCl pH 8.0, 200 mM NaCl, 0.2 mM phenyl methane sulfonyl fluoride). After centrifugation at 17 000 ×g for 1 h at 4 ℃, the supernatant was loaded to a Ni-NTA column (GE Healthcare), followed by a

washing buffer (25 mM Tris-HCl pH 8.0, 200 mM NaCl, 20 mM imidazole). The His-tagged AcFur and AcFur mutants were eluted with elution buffer (25 mM Tris-HCl pH 8.0, 100 mM NaCl, and 250 mM imidazole). Purified AcFur and AcFur mutants were analyzed by a 15% sodium dodecyl sulfate-polyacrylamide gel electrophoresis (SDS-PAGE) followed by coomassie brilliant blue R-250 staining.

Bioinformatics analysis of conservation of Fur proteins in the bacteria of AMD ecosystems

The evolutionary conservation structure and sequences of Fur were assessed using the ConSurf server(1). The ConSurf web servers calculated the evolutionary conservation of amino acid positions in the AcFur using an empirical Bayesian paradigm. The conservation scores were grouped into a nine grades scale and projected onto the structure and on the MSA for visualization.

Supplemental Figures

Fig. S1 Construction procedure of Δ*fur* and complementation strains. (A) Schematic representation of markerless gene knockout procedure. (B&C) Confirmation of Δ*fur* by PCR. Positions of verification primers used for PCR to confirm *fur* deletion. Lanes 1, 3, and 5, PCR amplifications from *A. caldus* MTH-04 wild type chromosome with primers F1/R1, F2/R2 and F3/R3, respectively. Lanes 2, 4, and 6, PCR amplifications from the *A. caldus* MTH-04 Δ*fur* chromosome with primers F1/R1, F2/R2 and F3/R3, respectively. (D&E) Confirmation of complementation strains by PCR. Positions of verification primers used for PCR to confirm complementation mutagenesis. Lanes 1, 2, 3, 4, 5,and 6, PCR amplifications with Δ*fur* (*fur*), Δ*fur* (SⅠ), Δ*fur* (SⅡ), Δ*fur* (SⅠ&Ⅱ), Δ*fur*, and WT chromosome, respectively. UHA and DHA represent upstream and downstream homologous arms, respectively.

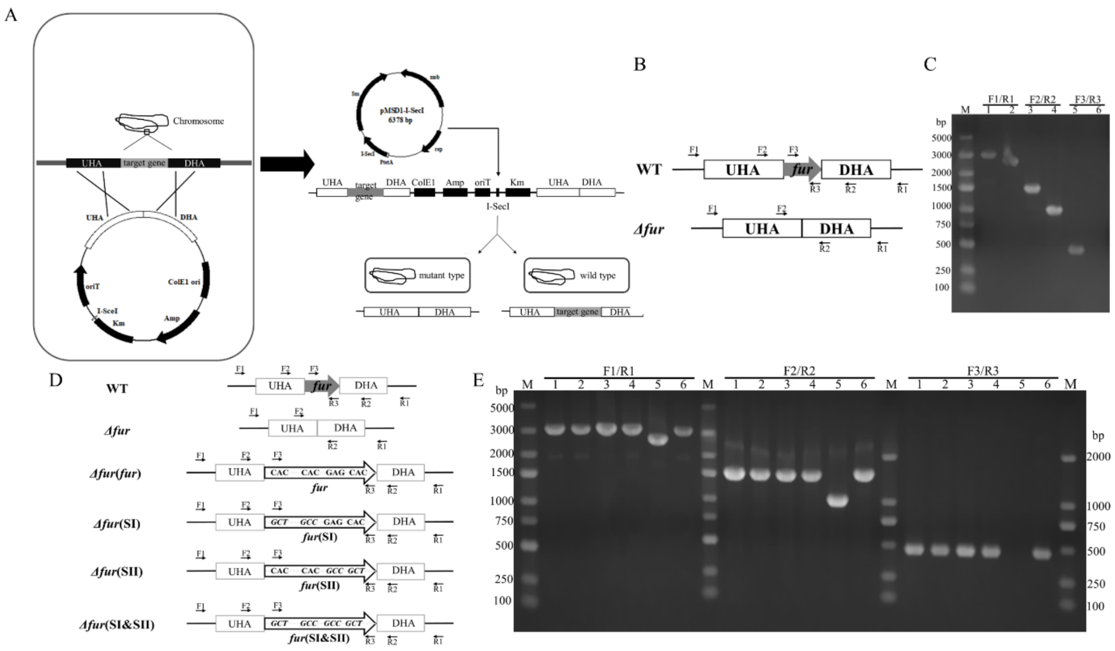

Fig. S2 Structure and sites conservation analysis of AcFur proteins and its homologs based on *Acidithiobacillus*. (A&B) The nine-color conservation grades are projected onto the structure and sequence of AcFur protein. The variable positions (grade 1) colored turquoise, the intermediately conserved positions (grade 5) colored white, the most conserved positions (grade 9) colored maroon.

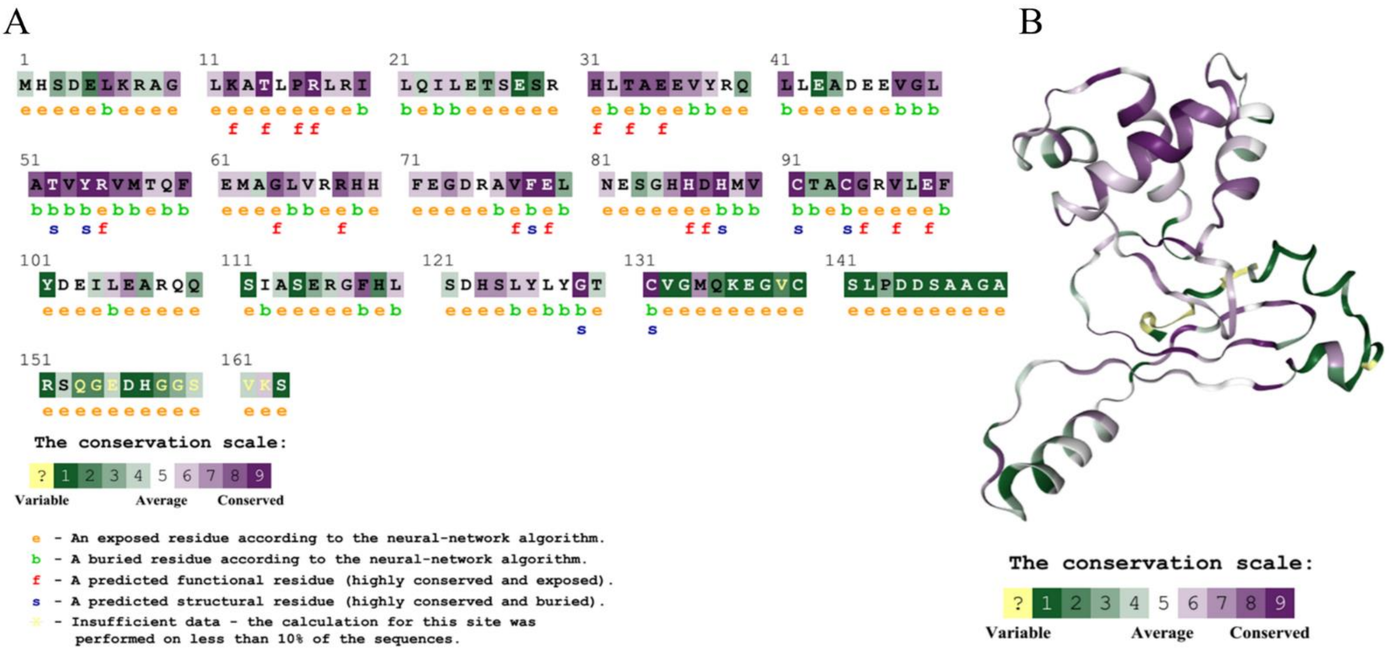

Fig. S3 Soluble test of Recombinant protein pET22b-dC. Recombinant protein pET22b-dC was pointed by red arrows. Lane M: M5 Prestained Protein Ladder Maker; lanes ce-s corresponding to the total proteins and supernatant of pET-22b; lanes ce-e corresponding to the crude extraction (ce), supernatant (s), flow (f),

washing (w), elution (e) of pET-22b-dC.

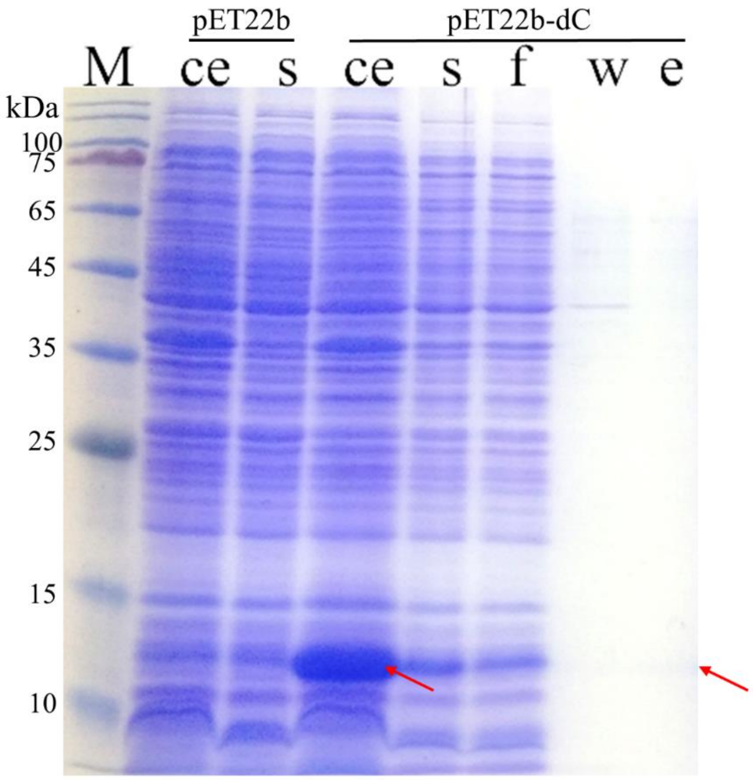

Supplemental Table

Table S1 Occurrence of fur and DtxR genes in published genomic information of AMD bacteria.

Phylum	Bacteria	Assembly level	Fur	DtxR
αProteobacteria	*Acidiphilium cryptum* JF-5	complete genome	NO	NO
	Acidiphilium multivorum AIU301	complete genome	NO	NO
	Acidiphilium angustum ATCC 35903	Scaffold	NO	NO
	Acidiphilium rubrum ATCC 35905	Scaffold	NO	NO
	Acidocella aminolytica 101	Scaffold	WP_048880410.1	NO
	Acidocella facilis ATCC 35904	Contig	WP_026439175.1	NO
	Acidomonas methanolica NBRC 104435	Scaffold	WP_081797598.1	NO
βProteobacteria	*Ferrovum myxofaciens* P3G	Contig	WP_031595537.1	NO
	Thiomonas intermedia K12	complete genome	WP_013121860.1	NO
	Thiomonas arsenitoxydans 3As	Chromosome	WP_013104191.1	NO
	Thiomonas delicata DSM 16361	Scaffold	WP_094159584.1	NO
	Thiomonas bhubaneswarensis DSM 18181	Scaffold	WP_055449419.1	NO

γProteobacteria	*Acidiferrobacter thiooxydans* ZJ	Contig	WP_083996317.1	NO
	Acidiferrobacter sp. SPIII_3	complete genome	WP_110138519.1	NO
	Acidihalobacter ferrooxidans V8	Chromosome	WP_076835706.1	NO
	Acidihalobacter prosperus F5	complete genome	WP_070077907.1	NO
	Metallibacterium scheffleri DKE6	Contig	WP_081127454.1	NO
Acidithiobacilla	*Acidithiobacillus thiooxidans* ATCC 19377	Scaffold	WP_010641352.1	NO
	Acidithiobacillus ferrooxidans ATCC 23270	complete genome	WP_012536062.1	NO
	Acidithiobacillus caldus	complete genome	WP_004869019.1	NO
	Acidithiobacillus ferrivorans SS3	complete genome	WP_014030069.1	NO
	Acidithiobacillus ferridurans JCM 18981	complete genome	WP_113526343.1	NO
	Acidithiobacillus albertensis DSM 14366	Scaffold	WP_010641352.1	NO
δproteobacteria	*Desulfomonile tiedjei* DSM 6799	complete genome	WP_014810043.1	WP_014809611.1
Acidobacteria	*Acidobacterium ailaaui* PMMR2	Contig	WP_026442842.1	WP_049961364.1
	Acidobacterium capsulatum ATCC 51196	complete genome	WP_015898090.1	ACO33208.1
	Granulicella mallensis MP5ACTX8	complete genome	WP_014263331.1	AEU38646.1
	Granulicella pectinivorans DSM 21001	Contig	WP_089840414.1	WP_089837736.1
	Granulicella rosea DSM 18704	Contig	WP_089407384.1	WP_142988166.1
	Granulicella tundricola MP5ACTX9	complete genome	WP_013572743.1	ADW70438.1
	Terriglobus saanensis SP1PR4	complete genome	ADV82922.1	ADV82279.1
	Terriglobus roseus DSM 18391	complete genome	NO	AFL86563.1
Nitrospirae	*Leptospirillum rubarum*	Scaffold	WP_014959897.1	NO
	Leptospirillum ferriphilum ML-04/YSK	complete genome	WP_014959897.1	NO
	Leptospirillum ferrooxidans C2-3	complete genome	WP_014448348.1	NO
	Leptospirillum sp. Group II 'CF-1'	complete genome	AKS24796.1	NO
	Leptospirillum sp. Group II 'C75'	Scaffold	EIJ75953.1	NO

	Leptospirillum ferrodiazotrophum	/	EES52611.1	NO
	Leptospirillum sp. Group IV 'UBA BS'	Scaffold	NO	NO
Actinobacteria	*Acidimicrobium ferrooxidans* DSM 10331	complete genome	ACU53263.1	NO
	Acidithrix ferrooxidans Py-F3	Contig	WP_052604974.1	NO
	Candidatus Koribacter versatilis Ellin345	complete genome	ABF42368.1	NO
	Candidatus Solibacter usitatus Ellin6076	complete genome	ABJ82847.1	ABJ83168.1
	Ferrimicrobium acidiphilum DSM 19497	Scaffold	KJE75465.1	NO
	Ferrithrix thermotolerans DSM 19514	Scaffold	SHE49700.1	NO
Firmicutes	*Sulfobacillus benefaciens*	Scaffold	PSR32246.1	NO
	Sulfobacillus acidophilus DSM 10332	complete genome	AEJ38577.1	NO
	Sulfobacillus thermosulfidooxidans DSM 9293	Scaffold	WP_051351132.1	NO
	Sulfobacillus sp. Hq2	Scaffold	WP_103375064.1	NO
	Sulfobacillus thermotolerans Kr	/	AUW95357.1	NO
	Alicyclobacillus acidiphilus NBRC 100859	Contig	WP_067621411.1	WP_067621532.1
	Alicyclobacillus acidocaldarius DSM 446	complete genome	ACV59616.1	WP_012811361.1
	Alicyclobacillus acidoterrestris ATCC 49025	Contig	EPZ52565.1	EPZ52800.1
	Alicyclobacillus macrosporangiidus DSM 17980	Scaffold	WP_074951538.1	WP_074955662.1
	Alicyclobacillus mali NBRC 102425	Contig	WP_067846234.1	WP_067845958.1
	Alicyclobacillus herbarius DSM 13609	Scaffold	WP_026961358.1	WP_026960854.1
	Alicyclobacillus contaminans DSM 17975	Scaffold	WP_026975363.1	WP_035485231.1
	Alicyclobacillus hesperidum	Scaffold	NO	WP_006446406.1
	Alicyclobacillus montanus USBA-GBX-503	Scaffold	WP_072873733.1	WP_072872655.1
	Alicyclobacillus ferrooxydans TC-34	Contig	WP_054968265.1	WP_054970710.1
	Alicyclobacillus shizuokensis NBRC 103103	Contig	WP_067927130.1	WP_067923196.1

	Alicyclobacillus sendaiensis NBRC 100866	Contig	WP_062305267.1	WP_062307455.1
	Alicyclobacillus kakegawensis NBRC 103104	Contig	WP_067932739.1	WP_067930834.1
	Alicyclobacillus tengchongensis	Contig	NO	WP_006446406.1
	Alicyclobacillus sacchari DSM 17974	Scaffold	WP_134158132.1	WP_134158038.1
	Alicyclobacillus pomorum DSM 14955	Scaffold	WP_026964647.1	NO
	Alicyclobacillus vulcanalis DSM 16176	Scaffold	WP_143232488.1	WP_076348602.1
	Desulfosporosinus acidiphilus SJ4	complete genome	AFM41980.1	NO
	Desulfosporosinus youngiae DSM 17734	Chromosome	EHQ90093.1	NO
	Desulfosporosinus orientis DSM 765	complete genome	AET68842.1	NO
	Desulfosporosinus lacus DSM 15449	Scaffold	SHH87716.1	SHI82465.1
	Desulfosporosinus hippei DSM 8344	Scaffold	SDG84826.1	NO
	Desulfosporosinus meridiei DSM 13257	complete genome	AFQ44521.1	NO
	Desulfosporosinus fructosivorans 63.6F	Scaffold	WP_135548847.1	WP_135550222.1
Aquificae	*Hydrogenobaculum sp.*	complete genome	WP_012513885.1	NO
Euryarchaeota	*Thermoplasma acidophilum* DSM 1728	complete genome	NO	DtxR 4O6J_A
	Thermoplasma volcanium GSS1	complete genome	BAB59437.1	BAB60108.1
	Thermoplasma sp. Kam2015	Contig	WP_110642587.1	WP_110641559.1
	Cuniculiplasma divulgatum PM4	complete genome	WP_021789929.1	NO
	Picrophilus oshimae DSM 9789	Contig	NO	SMD31358.1
	Picrophilus torridus DSM 9790	complete genome	NO	AAT43733.1
	Acidiplasma aeolicum	Contig	NO	WP_048101435.1
	Acidiplasma cupricumulans	Contig	NO	WP_048101435.1
	Acidiplasma sp. MBA-1	Contig	NO	WP_048101435.1
	Ferroplasma acidarmanus fer1	complete genome	NO	AGO60290.1
	Ferroplasma acidiphilum	complete genome	NO	WP_081142639.1
	Ferroplasma thermophilum	/	NO	NO
	Ferroplasma cyprexacervatum	Contig	NO	WP_048101435.1

	Ferroplasma cupricumulans	Contig	NO	WP_048101435.1
Crenarchaeota	*Acidianus hospitalis* W1	complete genome	NO	AEE93435.1
	Acidianus sulfidivorans JP7	complete genome	NO	AWR97065.1
	Acidianus brierleyi DSM 1651	complete genome	NO	WP_110270607.1
	Acidianus manzaensis YN-25	complete genome	NO	ARM75708.1
	Candidatus Acidianus copahuensis	Contig	NO	WP_048100246.1
	Acidilobus saccharovorans 345-15	complete genome	NO	NO
	Acidilobus sp. 7A	complete genome	NO	NO
	Metallosphaera sedula	complete genome	NO	WP_012021943.1
	Metallosphaera hakonensis JCM 8857	complete genome	NO	AWR99707.1
	Metallosphaera yellowstonensis	complete genome	NO	WP_048088038.1
	Metallosphaera cuprina Ar-4	Scaffold	NO	AEB94380.1
	Metallosphaera prunae	complete genome	NO	QCO30441.1
	Sulfolobus islandicus	Complete	NO	WP_012711465.1
	Sulfolobus acidocaldarius	Complete	NO	WP_011277038.1
	Sulfolobus metallicus	Contig	NO	WP_054838202.1
	Sulfurisphaera tokodaii	complete genome	NO	WP_010980111.1
	Saccharolobus solfataricus P2	complete genome	NO	AAK40972.1

References

1. Ashkenazy H, Erez E, Martz E, Pupko T, Ben-Tal N. 2010. ConSurf 2010: calculating evolutionary conservation in sequence and structure of proteins and nucleic acids. Nucleic Acids Res 38:W529-33.
